# Supplementary material for: Oral antigen exposure under costimulation blockade induces Treg cells to establish immune tolerance
Source: J Exp Med. 2025 Oct 21;223(3):e20251635. doi: 10.1084/jem.20251635 (PMC12539616; doi:10.1084/jem.20251635)
Supplement: Table S1 — shows nutrition facts of modified food. [file jem_20251635_tables1.docx]

**Table S1. Nutrition facts of modified food**

CE-2 (Normal Chow; NC)

| Nutrition facts | (%) |
| --- | --- |
| Water | 8.84 |
| Crude protein | 25.48 |
| Crude fat | 4.61 |
| Crude fiber | 5.14 |
| Crude ash | 7.01 |
| Nitrogen free extract | 48.92 |
| Total | 100.00 |

CE-2 (–)White fish powder (0%EWP)

| Nutrition facts | (%) |
| --- | --- |
| Water | 9.00 |
| Crude protein | 19.86 |
| Crude fat | 4.26 |
| Crude fiber | 5.30 |
| Crude ash | 6.24 |
| Nitrogen free extract | 55.34 |
| Total | 100.00 |

Modified food

| ID | CE-2 (–)White fish powder (%) | Corn starch (%) | EWP (%) | EWP derived protein (%) |
| --- | --- | --- | --- | --- |
| 0%EWP | 100 | 0 | 0 | 0 |
| 0.1%EWP | 99.89961 | 0.07743 | 0.02296 | 0.10000 |
| 1%EWP | 98.99610 | 0.77431 | 0.22959 | 1.00000 |
| 10%EWP | 89.96105 | 7.74300 | 2.29595 | 10.00000 |
| 20%EWP | 79.92200 | 15.48609 | 4.59191 | 20.00000 |
| 30%EWP | 69.88305 | 23.22909 | 6.88786 | 30.00000 |
