# Supplementary material for: Oral antigen exposure under costimulation blockade induces Treg cells to establish immune tolerance
Source: J Exp Med. 2025 Oct 21;223(3):e20251635. doi: 10.1084/jem.20251635 (PMC12539616; doi:10.1084/jem.20251635)
Supplement: Table S2 — shows the list of antibodies. [file jem_20251635_tables2.docx]

**Table S2. List of antibodies**

Antibodies used for flowcytometry, FACS sorting, and other experiments

| **Antibodies** | **Manufacturer** | **Identifier** |
| --- | --- | --- |
| Anti-mouse CD3e-APC (145-2C11) | BD pharmingen | Cat#553066; RRID: AB_398529 |
| Anti-mouse CD3e-APC-Cy7 (145-2C11) | BioLegend | Cat#100330; RRID: AB_1877170 |
| Anti-mouse CD3e-Biotin (145-2C11) | BD Biosciences | Cat#553060; RRID: AB_394593 |
| Anti-mouse CD3e-PE (145-2C11) | Invitrogen | Cat#12-0031-82; RRID: AB_465496 |
| Anti-mouse CD3e-V500 (500A2) | BD Biosciences | Cat#560771; RRID: AB_1937314 |
| Anti-mouse CD4-AF700 (RM4-5) | Invitrogen | Cat#56-0042-82; RRID: AB_494000 |
| Anti-mouse CD4-V500 (RM4-5) | BD Biosciences | Cat#560782; RRID: AB_1937327 |
| Anti-mouse CD4-BV650 (RM4-5) | BD Biosciences | Cat#563747; RRID: AB_2716859 |
| Anti-mouse CD8a-PerCP-Cy5.5 (53-6.7) | BD Biosciences | Cat#551162; RRID: AB_394081 |
| Anti-mouse CD11b-AF700 (M1/70) | Invitrogen | Cat#56-0112-80; RRID: AB_657585 |
| Anti-mouse CD11b-APC (M1/70) | BioLegend | Cat#101212; RRID: AB_312795 |
| Anti-mouse CD11b-PerCP-Cy5.5 (M1/70) | Invitrogen | Cat#45-0112-82; RRID: AB_468714 |
| Anti-mouse CD11c-BV605 (N418) | BIoLegend | Cat#117334; RRID: AB_2562415 |
| Anti-mouse CD11c-PE-Cy7 (N418) | BioLegend | Cat#117318; RRID: AB_493568 |
| Purified anti-mouse CD16/32 (93) | BioLegend | Cat#101302; RRID: AB_312801 |
| Anti-mouse CD25-BV421 (PC61) | BioLegend | Cat#102034; RRID: AB_11203373 |
| Anti-mouse CD25-PE (PC61) | BD pharmingen | Cat#553866; RRID: AB_395101 |
| Anti-mouse CD25-PE-Cy7 (PC61) | BD Biosciences | Cat#552880; RRID: AB_394509 |
| Anti-mouse CD25-PerCP-Cy5.5 (PC61) | BD Biosciences | Cat#551071; RRID: AB_394031 |
| Anti-mouse CD44-APC (IM7) | Invitrogen | Cat#17-0441-83; RRID: AB_469390 |
| Anti-mouse CD44-PE-Cy7 (IM7) | Invitrogen | Cat#25-0441-82; RRID: AB_469623 |
| Anti-mouse CD44-V500 (IM7) | BD Biosciences | Cat#560780; RRID: AB_1937328 |
| Anti-mouse CD45-BV510 (30-F11) | BioLegend | Cat#103138; RRID: AB_2563061 |
| Anti-mouse CD45.1-APC-Cy7 (A20) | BioLegend | Cat#110716; RRID: AB_313505 |
| Anti-mouse/human CD45R/B220-APC-eFluor780 (RA3-6B2) | Invitrogen | Cat#47-0452-82; RRID: AB_1518810 |
| Anti-mouse/human CD45R/B220-Biotin (RA3-6B2) | BioLegend | Cat#103204; RRID: AB_312989 |
| Anti-mouse CD62L-BV421 (MEL-14) | BD Biosciences | Cat#562910; RRID: AB_2737885 |
| Anti-mouse CD62L-PerCP-Cy5.5 (MEL-14) | BD pharmingen | Cat#560513; RRID: AB_10611578 |
| Anti-mouse CD64-PE (X54-5/7.1) | BioLegend | Cat#139304; RRID: AB_10612740 |
| Anti-mouse CD90.1-BV605 (OX-7) | BD Biosciences | Cat#740374; RRID: AB_2740106 |
| Anti-mouse CD90.1-PerCP (OX-7) | BD Biosciences | Cat#557266; RRID: AB_396611 |
| Anti-mouse CD90.2-AF700 (53-2.1) | BioLegend | Cat#140324; RRID: AB_2566740 |
| Anti-mouse CD90.2-PE-Cy7 (53-2.1) | BioLegend | Cat#140310; RRID: AB_10643586 |
| Anti-mouse CD101-APC (REA301) | Miltenyi Biotec | Cat#130-104-304; RRID: AB_2654344 |
| Anti-mouse CD101-PE (REA301) | Miltenyi Biotec | Cat#130-120-173; RRID: AB_2752032 |
| Anti-mouse CD101-PE-Vio770 (REA301) | Miltenyi Biotec | Cat#130-104-305; RRID: AB_2654345 |
| Anti-mouse CD103-APC (2E7) | Invitrogen | Cat#17-1031-82; RRID: AB_1106992 |
| Anti-mouse CD103-PE-Cy7 (2E7) | BioLegend | Cat#121426; RRID: AB_2563691 |
| Anti-mouse CD134(OX40)-BV421 (OX-86) | BioLegend | Cat#119411; RRID: AB_10962569 |
| Anti-mouse CD134(OX40)-PE (OX-86) | BioLegend | Cat#119410; RRID: AB_2207344 |
| Anti-mouse CD137(4-1BB)-APC (17B5) | BioLegend | Cat#106110; RRID: AB_2564297 |
| Anti-mouse CD195(CCR5)-BV421 (C34-3448) | BD Biosciences | Cat#743695; RRID: AB_2741677 |
| Anti-mouse CD199(CCR9)-PE (CW-1.2) | BioLegend | Cat#128710; RRID: AB_1227479 |
| Anti-mouse CD278(ICOS)-PE-Cy7 (C398.4A) | BioLegend | Cat#313520; RRID: AB_10643411 |
| Anti-mouse CD304(Neuropilin-1)-eFluor450 (3DS304M) | Invitrogen | Cat#48-3041-82; RRID: AB_2574051 |
| Anti-mouse CD304(Neuropilin-1)-PE-Cy7 (3DS304M) | Invitrogen | Cat#25-3041-82; RRID: AB_2573436 |
| Anti-mouse CD357(GITR)-PE-Cy7 (DTA-1) | BD Biosciences | Cat#558140; RRID: AB_647252 |
| Anti-mouse/human Helios-APC (22F6) | BioLegend | Cat#137218; RRID: AB_10660750 |
| Anti-mouse/human Helios-PE-Cy7 (22F6) | BioLegend | Cat#137236; RRID: AB_2565990 |
| Anti-mouse I-A/I-E-BV421 (M5/114.15.2) | BioLegend | Cat#107631; RRID: AB_10900075 |
| Anti-mouse I-A/I-E-BV510 (M5/114.15.2) | BioLegend | Cat#107635; RRID: AB_2561397 |
| Anti-mouse/human Ki-67-PE-Cy7 (SolA15) | Invitrogen | Cat#25-5698-82; RRID: AB_11220070 |
| Anti-mouse/human KLRG1-PerCP-Cy5.5 (2F1/KLRG1) | BioLegend | Cat#138418; RRID: AB_2563014 |
| Anti-mouse/human T-bet-BV421 (4B10) | BioLegend | Cat#644816; RRID: AB_2686976 |
| Anti-mouse TCR DO11.10-APC (KJ1-26) | Invitrogen | Cat#17-5808-80; RRID: AB_469459 |
| Anti-mouse TCR DO11.10-PerCP-Cy5.5 (KJ1-26) | BioLegend | Cat#118512; RRID: AB_2028522 |
| Anti-mouse TER-119/Erythroid Cells-Biotin (TER-119) | BD pharmingen | Cat#553672; RRID: AB_394985 |
| Anti-mouse TIGIT-APC (1G9) | BioLegend | Cat#142105; RRID: AB_10960139 |
| Anti-mouse Foxp3-Alexa Fluor 488 (FJK-16s) | Invitrogen | Cat#53-5773-82; RRID: AB_763537 |
| Anti-human/mouse Gata3-eFlour660 (TWAJ) | Invitrogen | Cat#50-9966-42; RRID: AB_10596663 |
| Anti-mouse RORgamma(t)-PE (B2D) | Invitrogen | Cat#12-6981-82; RRID: AB_10807092 |
| Anti-mouse IL-4-APC (11B11) | Invitrogen | Cat#17-7041-82; RRID: AB_469494 |
| Anti-mouse IL-17A-BV421 (TC11-18H10.1) | BioLegend | Cat#506925; RRID: AB_10900442 |
| Anti-mouse IFN gamma-PE (XMG1.2) | Invitrogen | Cat#12-7311-82; RRID: AB_466193 |
| TotalSeq-C0301 anti-mouse Hashtag 1 Antibody | BioLegend | Cat#155861; RRID: AB_2800693 |
| TotalSeq-C0302 anti-mouse Hashtag 2 Antibody | BioLegend | Cat#155863; RRID: AB_2800694 |
| TotalSeq-C0303 anti-mouse Hashtag 3 Antibody | BioLegend | Cat#155865; RRID: AB_2800695 |
| TotalSeq-C0304 anti-mouse Hashtag 4 Antibody | BioLegend | Cat#155867; RRID: AB_2800696 |
| Purified NA/LE anti-mouse CD3e (145-2C11) | BD pharmingen | Cat#553057; RRID: AB_394590 |
| Purified NA/LE anti-mouse CD28 (37.51) | BD pharmingen | Cat#553294; RRID: AB_394763 |
| Anti-Histone H3 Acetyl Lys27 | GeneTex | Cat#GTX60815; RRID: AB_2888004 |
